# Effects of a randomized controlled trial of a brief, student-nurse led, parent-based sexual health intervention on parental protective factors and HPV vaccination uptake

**DOI:** 10.1186/s12889-021-10534-0

**Published:** 2021-03-24

**Authors:** D. Santa Maria, C. Markham, S. M. Misra, D. C. Coleman, M. Lyons, C. Desormeaux, S. Cron, V. Guilamo-Ramos

**Affiliations:** 1 University of Texas Health Science Center at Houston, Cizik School of Nursing, 6901 Bertner Ave, Houston, TX USA; 2 Health Promotion and Behavioral Sciences, University of Texas Health Science Center at Houston School of Public Health, 7000 Fannin Street, Houston, TX USA; 3 Baylor College of Medicine, Texas Children’s Hospital, 8080 North Stadium Drive, Suite 250, Houston, TX USA; 4 Cizik School of Nursing, University of Texas Health Science Center at Houston, Houston, TX USA; 5 Center for Latino Adolescent and Family Health, New York University, New York, NY USA

**Keywords:** Human papillomavirus, HPV vaccine, Parent-child sexual health communication, Parental connectedness, Adolescent sexual behavior

## Abstract

**Background:**

Parents play a pivotal role in adolescent sexual health and Human Papillomavirus (HPV) vaccination. Nurses are on the frontlines of healthcare and play a critical role in promoting HPV vaccination and parent-child sexual health communication. We enhanced the *Families Talking Together* (FTT) parent-based sexual health curriculum to include adolescent vaccinations herein, FTT + HPV, and trained student nurses to provide a strong HPV vaccination and parent-child sexual health communication endorsement.

**Methods:**

Using a randomized attention-controlled trial design, we examined the efficacy of FTT + HPV among 519 parents and their 11–14 year old youth recruited from medically underserved communities between 2015 and 2018. Participants were recruited from 22 after-school programs (e.g., Boys and Girls Clubs) and 19 charter schools. For parents, we examined protective factors including parent-child sexual health communication and parental involvement. For youth, we examined sexual health knowledge, parent-child sexual health communication, and parent-child connectedness. To assess HPV vaccination initiation and completion, we searched IMMTRAC immunization registry records for 85% of youth and used parental report for youth without registry records. Group differences were calculated using the estimated mean difference at one- and six months post-intervention with significance set at the *p* < 0.05 level.

**Results:**

Baseline rates of HPV vaccination were low at 55.7%. No significant difference between the groups was seen in vaccination initiation or completion rates by one-month post-intervention. However, by six-months post intervention, there was a significant difference between the groups with 70.3% of the intervention group initiating the HPV vaccination series vs. 60.6% for the control group (*p* = 0.02). No difference between the groups was found for HPV series completion at six-months. There were significant differences in condom knowledge (*p* = 0.04), parent-child connectedness (*p* = 0.04), and communication frequency (*p* = 0.001) with greater improvement in the intervention vs. the control group. Rates of sexual activity remained low in both groups throughout the six-month follow-up period.

**Conclusion:**

A brief parent-based adolescent sexual health and HPV vaccination intervention delivered by student nurses can improve sexual health outcomes including protective parental factors, adolescent sexual health knowledge, and HPV vaccination initiation rates.

**Trial registration:**

ClinicalTrials.gov Identifier: NCT02600884. Prospectively r*egistered September 1, 2015.*

**Supplementary Information:**

The online version contains supplementary material available at 10.1186/s12889-021-10534-0.

## Background

Puberty is considered the beginning of human sexual development consisting of physical and emotional maturity marking the transition from childhood to adulthood. While engaging in sexual activity is a normal behavior, early sexual activity is associated with negative outcomes including unplanned pregnancy and sexual transmitted infections [1]. About 20% of 9th graders in the U.S. report being sexually active [18]. Early sexual debut is associated with inconsistent and nonuse of contraceptives and is a risk factor for sexually transmitted infections (STIs) and pregnancy [20, 22]. Each year in the U.S., adolescents account for nearly half of new STI cases (costing $6.5 billion) and experience about 750,000 pregnancies costing $11 billion [2, 15, 16, 38]. Adolescents experience the largest burden of STIs, HIV, and unplanned pregnancy in the U.S. [10, 25] with a birth rate of 18.8 per 1000 adolescents 15–19 years old in 2017 [24]. Human papillomavirus (HPV) continues to be the most common STI in both men and women in the U.S. [31]. Despite the Centers for Disease Control and Prevention (CDC) vaccination guidelines and the widespread availability of free vaccination programs for males and females, initiation and completion rates remain far below the CDC goal of 80% among early adolescents [33].

There are more than 100 different HPV viral types [31] with 80 million men and women currently infected with at least one type of HPV in the U.S. and 14 million Americans becoming newly infected every year [7]. The average lifetime probability of HPV is 84.6% for women and 91.3% for men, with more than 80% acquiring HPV by age 45 [3]. Of those infected with HPV, 4.9% were infected with high-risk strains known for causing cancer [26]. Additionally, disparities exist among racial and ethnic minorities in the U.S. with the prevalence of genital and oral HPV infection being higher in the non-Hispanic black population than Hispanic and White populations [26]. This is particularly important as Hispanic and non-Hispanic black women have the highest incidence rates of HPV-associated cancers than women in all other ethnic and racial groups. Disparities in HPV vaccination also exist. Among youth 13–17 years old in the 2008–2009 National Immunization Survey-Teen (*n* = 18,228), Hispanic and Black youth were less likely than White youth to complete the HPV series [9].

HPV vaccination completion rates vary across states with rates in Texas being among the lowest and remaining lower (39.7%) than the national average (48.6%) [37]. In addition to the benefits of state issued vaccination mandates [11], research also demonstrates that simple, cost effective methods such as communication and following vaccination guidelines at age specific visits for adolescents are effective at increasing vaccination rates. Lu et al. [21] found that vaccine recommendations by healthcare providers caring for adolescent males 13–17 increased HPV utilization in Texas [21]. Additionally, adolescents were more likely to initiate the HPV series when the vaccine was bundled with other recommended vaccine(s) [21] and when endorsed by their provider [28].

Parents play a pivotal role in adolescent sexual health and HPV vaccination of adolescents. Educational programs that target parents aim to reduce sexual risk behaviors among adolescents such as early sexual debut and condomless sex by increasing parental protective factors such as parent-child sexual health communication [39], parental monitoring [4], and HPV vaccination rates [8]. In a meta-analysis of parent-child sexual health interventions, intervention participants were 68% more likely than the control group to report increased communication (Cohen’s d, 0.5) and also 75% more likely to report increased comfort with sexual health communication (Cohen’s d, 0.7) [30]. These effects were positive regardless of delivery mode or intervention dose indicating that even brief interventions can also improve parental protective factors [30].

### Theoretical framework

This intervention aimed to reduce adolescent sexual risk behaviors and increase HPV vaccination by bolstering parental protective factors as demonstrated by Hutchinson’s parent-based expansion of the theory of planned behavior [19]. This framework posits that parents are more likely to discuss sexual health with youth if they have intentions to communicate, have positive beliefs about parent-child communication (behavioral beliefs), believe that others important to them approve of sexual health communication (normative beliefs), and feel that they have the skills needed (control beliefs) to effectively communicate about sexual health topics.

The purpose of the study was to evaluate the efficacy of a brief parent-based adolescent sexual health intervention on parental protective factors and HPV vaccination rates. The intervention was conducted by student nurse facilitators with parents of 11–14 year old youth from medically underserved communities.

## Methods

### Recruitment

Parents and caregivers of youth 11–14 years of age were recruited from 22 after-school programs (e.g. Boys and Girls Clubs) and 19 charter schools in medically underserved communities between 2015 and 2018. Written informed consent was received from the parent participants and written assent was received from the youth. All study procedures were approved from the Committee for the Protection of Human Subjects at the University of Texas Health Science Center at Houston (HSC-SN-15-0091) prior to recruitment and enrollment of study participants. Student research assistants enrolled youth as dyads with their parent and randomly assigned dyads using a computerized random number generator to either the intervention or control group using a 1:1 group allocation. At follow-up, the study team research assistants were blinded to group assignment. Parents and youth received $20 for each completed survey.

First, we used community-based participatory methods to adapt *Families Talking Together* (FTT) to include a module on adolescent vaccinations and HPV specifically. FTT + HPV has three main components: a brief face-to-face session, a take-home manual, and booster calls. FTT has been successful in delaying sexual debut in minority youth at nine months post-intervention when implemented in clinics and schools [13, 14]. Importantly, it is available in English and Spanish.

Nurses are the largest frontline healthcare provider workforce and well-positioned to deliver health promoting interventions in community settings. To build on this expertise, we wanted to assess the delivery of FTT + HPV by student nurses [29]. Therefore, we recruited undergraduate senior level student nurses from a public health nursing clinical course who received approximately 32 h of extensive training on the implementation of the FTT + HPV program and community-based research to serve as the interventionists. Training included Protection of Human Subjects certification, parent-based adolescent sexual health, STIs and HPV, HPV vaccination, and health education communication methods and strategies.

### Intervention description

In the *face-to-face session,* the parent and student nurse met for approximately 45 min to review the FTT + HPV materials, motivate parents to talk with their children, and address specific components of the program. Student nurses helped parents designate a time to talk with their children and reviewed information about the context of the present-day teen’s world (e.g., physical changes, teen thinking, peers, emotions, and teen moral development) and how a parent can help a teen through positive parenting (e.g., parenting styles, child discipline, parental monitoring, communication, relationship building, forming healthy relationships, self-esteem, refusal and negotiation skills, and risk reduction strategies). The student nurse reviewed information about adolescent vaccinations including the importance of the HPV vaccine, presented local resource materials detailing where and when the child can get vaccinated, and helped the parent make an appointment for vaccination when on-site vaccination clinics were available. Each parent received a manual that reiterated the above-mentioned information as well as three handouts to supplement the face-to-face session. The handouts discussed adolescent vaccinations, contraceptives, and healthy relationships. Parents were encouraged to work through the activities in the manual with their child over the following weeks.

The *manual* was divided into sections covering health and social consequences of premature sexual behaviors, positive parental influences on adolescent sexual behaviors, saying ‘no’ to sex, common teen beliefs about sex, monitoring and supervision strategies, parent-child relationship building, and communication tips. Two follow-up telephone-based *booster calls* were delivered at one- and three-months post-intervention. During the booster session call, the student nurse discussed the parent’s progress with communication and vaccination and discussed barriers they were facing while progressing through the manual with their child. Bilingual nursing students were assigned to participants who preferred to receive the intervention discussion or materials in Spanish. When possible, we coordinated with a local pediatric mobile vaccination clinic to offer all childhood vaccinations free of charge through the Vaccines for Children program during the recruitment events. A total of seven vaccination events were coordinated.

The attention *control group* parents received information from the student nurse on promoting healthy nutrition and exercise among adolescents in a 45-min session. During the session, the student nurse and the parent set a goal related to nutrition and physical activity for their child. Parents also received a brochure of healthy lifestyles and booster calls and 1- and 3-months post-intervention. Similarly, all materials and sessions were available in English and Spanish.

### Parent measures

Baseline surveys collected data about parents’ demographic characteristics including gender, race/ethnicity, level of education, parental role, annual household income, religiosity, and insurance status. In addition, a battery of psychosocial measures were collected at baseline, one, and six months. The primary behavioral outcomes of interest for parents were parent-child sexual health communication, parental intention to vaccinate their children for HPV, and vaccination uptake and completion rates. Psychosocial determinants known to influence parental practice, sexual health communication, and parental monitoring were secondary outcomes examined in this study, as well as factors associated with HPV vaccine uptake and completion such as vaccine beliefs. Parent communication expectancies associated with parent-youth discussions about sex was assessed using a 15-item scale developed by DiIorio et al. [5]. Each item begins with the stem, “*If you talk with your child about sex topics*...” and is followed by an expected outcome for parents such as, “*you will feel that you did the right thing*” or “*it would be unpleasant*” [5] with options from strongly disagree to strongly agree on a 5-point Likert scale. Parent communication self-efficacy was assessed using a 16-item scale. Sample items from the scale include, “*You can always explain to your child what you think about adolescents their age having sex*” [6]. Response options ranged from “not sure at all” to “completely sure” on a 7-point Likert scale. Frequency of communication about sex was assessed using an 8-item scale [6]. A sample item is: “*In the past month, how often have you talked to your child about how to handle sexual pressure by friends or potential partners?*” with a 10-point scale ranging from never to 10 times or more. Communication ability was assessed using a single item: “*How would you rate your ability to communicate with your child about sexual topics?*” [6] on a 7-point scale. Communication openness was defined as the extent to which parents feel comfortable talking to their child about sex and was assessed using an 8-item scale [14] with response options ranged from strongly agree to strongly disagree on a 4-point scale.

Secondary parental outcomes included parental connectedness which was defined as the degree of closeness between a parent and child using a 4-item scale [14]. Parents were asked to respond to the following statement: “*Most of the time, your child is warm and loving toward you*.” Response options ranged from not at all to very much on a 1–5 scale. Parental involvement, actively participating in a child’s life, was assessed using a 10-item scale [14]. A sample item is, “*During the past month, how many times did you and your child do fun things together?*” Response options were on a 5-point scale that ranged from “not at all” to “7 or more times.” Parental monitoring was assessed using a 7-item scale [14]. A sample item is: “*Do you currently have clear rules or expectations about where your child can go after school?*”

Parental vaccine beliefs were assessed using a 22-item scale. Sample items include “*Vaccinations protect children from getting diseases from unvaccinated children*” and “*I am more likely to trust vaccinations that have been around awhile*.” Response options ranged from disagree to agree on a 5-point scale. Intention to vaccinate was assessed using a single item: “*Are you planning to give your child all 3 doses of the HPV series?*” This item was scored on a dichotomous scale (0 = no, 1 = yes).

Participant scores for the parent measures were calculated as the mean of the answered items multiplied by the total number of items in the scale. This method addresses missing data by using the scale items that were answered by a participant to calculate their scale score (Newman, 2014). Cronbach’s alpha for the parent measures ranged from .74 to .93.

### Youth measures

At baseline, gender, age, and race/ethnicity were among the sociodemographic variables collected for youth. In addition, a host of behavioral and psychosocial measures were administered at baseline, one- and six-months.

The primary outcomes of interest for youth were sexual activity and HPV vaccine uptake. Youth self-reported whether they had ever engaged in oral, vaginal, or anal sex at baseline, one, and six months. These items were scored on a dichotomous scale (0 = no, 1 = yes). Youth also reported frequency of sexual behavior in the past month which was scored on a 6-point Likert scale ranging from never to more than 10 times. To assess HPV vaccine initiation and completion, vaccination records were obtained from IMMTRAC, a state-wide vaccination registry. Records were available for most youth in the study (85%); parental report was used for the 15% of youth whose vaccination records were not listed in the registry.

Secondary outcomes known to influence adolescent sexual behavior we also assessed.

Beliefs about sex were assessed using a 4-item scale [34]. A sample item is: “*I believe people my age should wait until they are older to have sex*.” Beliefs about abstinence were examined with a 6-item scale [34]. For example, youth were asked to respond to the following statement: “*The best way for young people to avoid an unwanted pregnancy is to wait until they are married before they have sex*.” Perceived parents beliefs about sex were assessed with a 4-item scale [34]. A sample item from the scale is “*My caregiver believes people my age should wait until they are older before they have sex*.” Response options for these 3 scales ranged from strongly agree to strongly disagree on a 1–4 scale.

Self-efficacy for refusing sex was examined using a 7-item scale [6]. Each item begins with the stem “*Could you stop the person that you like from…”* which is followed by intimate behaviors such as “*kissing you on the lips, if you did not want them to do that*” or “*touching your private parts below the waist, if you did not want them to do that*.” Response options were on a 4-point scale and ranged from definitely could not to definitely could. Condom knowledge was assessed with a 6-item scale [32]. Sample items include: “*Do condoms help keep a person from getting pregnant*?” with response options of “Yes,” “No” or “Not sure.” Condom self-efficacy was assessed using a 4-item scale [34]. A sample is: “*How sure are you that you could tell your partner you want to use condoms*?” Youth responded on a 1–3 scale ranging from “not at all sure” to “definitely sure.” *Exposure to risky situations* was assessed using a 6-item scale [6]. Each item begins with the stem “*In the past month, how often have you….”* and is followed by a scenario which might make a youth vulnerable such as “gone to, or stayed at, a party where alcohol was being used”. Response options ranged from “never” to “daily” on a 7-point scale.

HIV/STI knowledge was assessed with a 5-item scale [6]. A sample is: “*You can tell if a person has HIV or AIDS just by looking at them.*” Response options for youth were true, false, or not sure. Intentions towards sex were examined using a 5-item scale [6]. Each item begins with the stem “*How likely is it that you will….”* and is followed by statements such as “*have oral sex in the next year*” or “*remain sexually abstinent from now until the marriage*” on a 1–5 scale. Expectancies for youth were assessed using a 21-item scale that inquired about how the youth would feel if he/she had sexual intercourse at this point in life and included “*it would be embarrassing for me if I got pregnant or got a girl pregnant*” or “*I would feel more attractive”* using a 5-point scale from agree to disagree [6].

Communication self-efficacy can be defined as a youth’s level of comfort with talking to their parent about sex and was assessed using a 16-item scale [14]. Each item begins with the stem “*How sure are you that you can talk to your caregiver about*…” followed by topics like “*where to buy or get condoms*” or “*how to tell a boy/girl no if you do not want to have sex*” on a 1–4 scale. Communication about sex outcome expectancy was assessed using a 15 item-scale [6]. A sample item was “*If you talk with your caregiver about sex topics you will feel responsible*” using a 5-point scale from strongly disagree to strongly agree. Communication about sex was assessed with 8-items examining the content of parent-youth sexual health discussions [17]. Youth were asked questions like “Ha*ve you ever talked to your caregiver about when to start having sex”* with response options of yes and no. Communication ability was examined using a single item from Schuster et al. [32]: *How would you rate your ability to communicate with your caregiver about sexual topics* [32]*?* Youth responded on a 7-point scale ranging from excellent to terrible. Communication content and frequency was assessed by 21-items examining parent-youth sexual health communication [6]. Each item begins with the stem “*How many times has your caregiver ever talked to you about…*” followed by topics such as “*how you will make decisions about whether to have sex*” on a 1–5 scale ranging from never to 10 times or more.

Parent-youth connectedness was assessed using a 5-item scale [14]. Sample items include: “*How close do you feel to your caregiver*?” with response options from 1 to 5. Parental monitoring was examined using 5-items that measured youths’ perception of parents’ knowledge about what the youth is doing, who they are with, and where they are in their free time [14]. A sample item is: “*How much does your caregiver know about who your friends really are*?” with responses on a 4-point scale. Intentions and beliefs about child disclosure about sex was assessed using a 4-item scale. A sample is: “*I plan to talk to my caregiver about sexual health issues in the future*” with response options on a 5-point scale.

Participant scores for the youth measures were also calculated as the mean of the answered items multiplied by the total number of items in the scale. Cronbach’s alpha for the youth measures ranged from .71 to .95, except for the intentions towards sex scale, which had a reliability measure of .63.

### Statistical analyses

#### Sample size

The primary research question is the effectiveness of FTT+ in decreasing the proportion of students initiating sexual activity. A sample size (530 adolescent-parent dyads) was calculated to ensure adequate power to test for differences between the intervention and control groups in sexual activity at 6 months. We estimate a participation rate of 85% or higher based on anticipated community support and the use of incentives. Assuming an attrition rate of 15%, a final sample size of 450 was indicated as needed. Based on this sample size, a chi-square test will have 80% power when the effect size is h = .27, or when the proportion of intervention and control participants engaging in sexual activity at 6 months is .12 and .22, respectively.

Descriptive statistics were calculated for demographic variables and instrument scores at each time point. Reliability estimates of the instruments were computed with Cronbach’s alpha. Inferential statistical analysis was based on the intention-to-treat principle and included all participants who were randomly assigned. The chi-square test was used to compare the groups for sexual behavior and HPV vaccination at each time point. Repeated measures analysis with linear mixed models was used to compare the groups for change over time in the mean instrument scores.

Linear mixed models include all participants with one or more observations in the analysis, with estimates of change in the outcomes based on all observable data. This increases power by allowing for all available data to be included in the analysis. In addition, this reduces bias by including all available data from those that were lost to follow-up in the calculations of the estimates. Linear mixed models were also used to conduct sensitivity analysis to assess the impact of missing data through loss to follow-up (see supplemental materials). These models did not show significant differences for change in parental and youth outcomes due to loss to follow-up (all *p* > .18).

Logistic regression models were used to test if the interaction of parental communication and parental monitoring was associated with youth sexual behavior at six months. As three parental communication measures were collected, separate models were tested for each. Statistical analyses were conducted with SAS 9.4 for Windows. Group differences were calculated using the estimated mean difference at one- and six-months post intervention with significance set at the *p* < 0.05 level. This study adheres to CONSORT guidelines and includes a completed CONSORT checklist as an additional file.

## Results

**Fig. 1:**
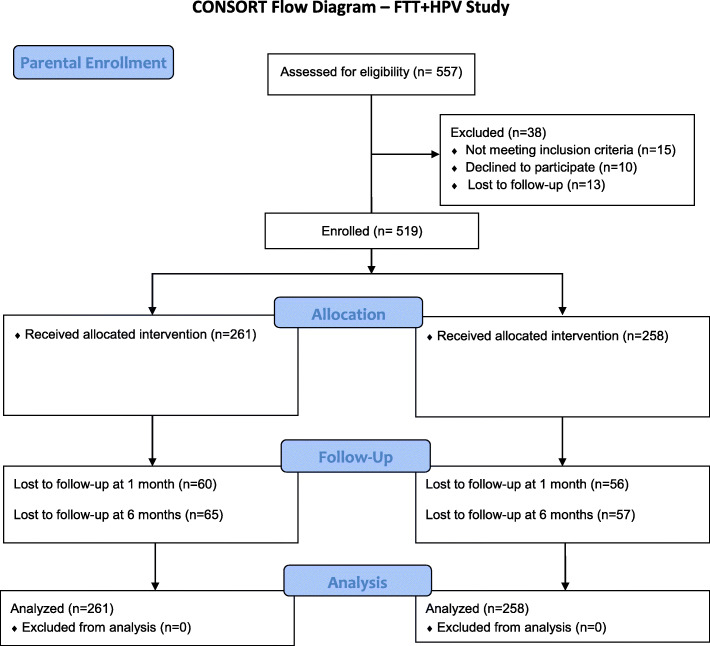
CONSORT Flow Diagram Parents – FTT + HPV Study

**Fig. 2:**
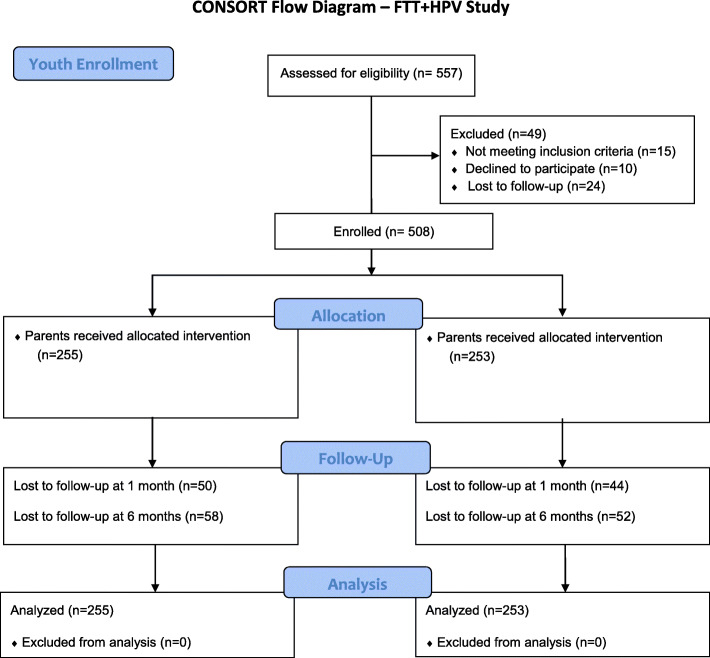
CONSORT Flow Diagram Youth – FTT + HPV Study

We screened 557 parent/child dyads for this study of which 519 parents and 508 youth completed the baseline survey and fully enrolled (see Figs. 1 and 2). At 1-month follow-up, 116 parents and 94 youth were lost to follow-up (i.e. unable to contact participant or complete the survey on time). At 6-months, 122 parents and 111 youth were lost to follow-up.

### Sample characteristics

Parents (*n* = 519) were primarily female (90.3%), Hispanic (56.8%) or Black (38.3%), and had some college education (44.9%) (Tables 1 and 2). The majority of parents identified as mothers (81.9%), though fathers (5.6%) and grandparents (4.1%) also participated with the remaining caregivers identifying as foster, adoptive, and step-parents. There were no differences in parental role distribution among the intervention and control groups. The largest proportion of participants had Medicaid (42.1%) with 15% indicating they had no insurance coverage. Youth (*n* = 508) were on average 12.6 years old, 50.8% female, and primarily youth of color (54.3% Hispanic, 40.9% Black). Most youth were in either 6th (30.7%) or 7th (27.1%) grade at baseline.

**Table 1:** Parent Baseline Socio-Demographic Characteristics by Condition

Characteristic	Total Parent Sample	Parent Intervention Group	Parent Control Group	*P* Value
Sample Size	519	261	258	
Gender				.74
Male	50 (9.73%)	24 (9.30%)	26 (10.16%)	
Female	464 (90.27%)	234 (90.70%)	230 (89.84%)	
Race/Ethnicity				.56
African American	199 (38.34%)	102 (39.08%)	97 (37.60%)	
Hispanic	295 (56.84%)	147 (56.32%)	148 (57.36%)	
White	14 (2.70%)	5 (1.92%)	9 (3.49%)	
Other	11 (2.12%)	7 (2.68%)	4 (1.55%)	
Education				.20
Did Not Finish High School	96 (18.71%)	53 (20.54%)	43 (16.86%)	
High School Graduate	121 (23.59%)	61 (23.64%)	60 (23.53%)	
Vocational/Technical	66 (12.87%)	32 (12.40%)	34 (13.33%)	
Some College	119 (23.20%)	63 (24.42%)	56 (21.96%)	
College Graduate	111 (21.64%)	49 (18.99%)	62 (24.31%)	
Insurance Status				.36
None	81 (15.70%)	44 (16.92%)	37 (14.45%)	
Medicaid	217 (42.05%)	116 (44.62%)	101 (39.45%)	
Private	168 (32.56%)	76 (29.23%)	92 (35.94%)	
Other	50 (9.69%)	24 (9.23%)	26 (10.16%)

**Table 2:** Youth Baseline Socio-Demographic Characteristics by Condition

Characteristic	Total Youth Sample	Youth Intervention Group	Youth Control Group	*P* Value
Sample Size	508	255	253	
Mean Age (sd)	12.57 (1.17)	12.58 (1.22)	12.57 (1.11)	.88
Gender				.25
Male	247 (49.20%)	117 (46.61%)	130 (51.79%)	
Female	255 (50.80%)	134 (53.39%)	121 (48.21%)	
Race/Ethnicity				.46
African American	208 (40.94%)	112 (43.92%)	96 (37.94%)	
Hispanic	276 (54.33%)	131 (51.37%)	145 (57.31%)	
White	12 (2.36%)	5 (1.96%)	7 (2.77%)	
Other	12 (2.36%)	7 (2.75%)	5 (1.98%)	
Attending School				1.0
Yes	498 (99.40%)	249 (99.20%)	249 (99.60%)	
No	3 (0.60%)	2 (0.80%)	1 (0.40%)	
Current Grade				.56
4th	5 (1.00%)	4 (1.59%)	1 (0.40%)	
5th	85 (16.93%)	51 (20.32%)	34 (13.55%)	
6th	154 (30.68%)	69 (27.49%)	85 (33.86%)	
7th	136 (27.09%)	63 (25.10%)	73 (29.08%)	
8th	99 (19.72%)	48 (19.12%)	51 (20.32%)	
9th	23 (4.58%)	16 (6.37%)	7 (2.79%)	

### Parent outcomes

Parent-child sexual health communication expectancy, self-efficacy, frequency, ability, and openness were analyzed for intervention effects on the change in mean score between intervention and control participants (Table 3). Improvements in parental communication frequency was found at one- and six-months post intervention though no significant difference in expectancy, self-efficacy, ability, or openness. A significant intervention effect was found for communication frequency with a larger mean score increase in frequency among the intervention group (*p* = 0.001). While there was no significance difference in parent reported parent-child connectedness, there were significant differences in parental involvement between the groups with a higher mean score increase found among intervention parents (*p* = 0.02). No significant differences were found for other parental reported communication measures including parent child sexual health communication expectancy, parent-child communication self-efficacy, communication ability, or openness. There were also no significant intervention effect on parental monitoring.

**Table 3:** Parent Child Communication, Monitoring, Connectedness, and HPV Vaccine Beliefs by Condition and Assessment Period (Means and Standard Deviations)

Parental Outcomes				
Variable	Baseline (*n* = 519)	1-Month (*n* = 403)	6-Month (*n* = 397)	*P* Value^1^
Primary Parent Outcomes				
Parent Communication Expectancy				.85
Intervention Group	57.27 (7.83)	57.93 (7.44)	58.16 (8.64)	
Control Group	57.58 (8.16)	57.75 (8.30)	58.18 (8.75)	
Parent Communication Self-Efficacy				.21
Intervention Group	89.01 (17.87)	93.02 (16.05)	95.92 (15.41)	
Control Group	90.15 (18.17)	93.82 (15.26)	93.98 (16.34)	
Frequency of Communication				.001
Intervention Group	17.18 (13.54)	23.89 (16.54)	25.89 (17.77)	
Control Group	18.09 (14.86)	20.67 (15.28)	21.63 (17.05)	
Communication Ability				.49
Intervention Group	2.90 (1.41)	2.49 (1.19)	2.41 (1.21)	
Control Group	2.92 (1.50)	2.70 (1.32)	2.57 (1.33)	
Communication Openness				.19
Intervention Group	17.10 (4.84)	15.09 (4.14)	14.90 (4.13)	
Control Group	16.88 (4.57)	15.74 (4.06)	15.10 (4.66)	
Secondary Parent Outcomes				
Parent-Child Connectedness				.65
Intervention Group	17.82 (2.74)	17.96 (3.00)	18.16 (2.73)	
Control Group	17.35 (2.87)	17.28 (3.06)	17.63 (2.90)	
Parental Involvement				.02
Intervention Group	35.28 (5.27)	36.29 (4.72)	36.77 (4.56)	
Control Group	35.64 (5.55)	35.56 (5.63)	36.00 (5.36)	
Parental Monitoring				.45
Intervention Group	7.34 (0.87)	7.35 (0.99)	7.24 (0.80)	
Control Group	7.42 (1.09)	7.32 (0.96)	7.23 (0.75)	
HPV Vaccine Beliefs				.64
Intervention Group	82.33 (11.68)	84.08 (11.22)	84.03 (12.62)	
Control Group	82.64 (11.59)	83.67 (11.04)	83.35 (11.54)	
Secondary Youth Outcomes				
Variable	Baseline (*n* = 508)	1-Month (*n* = 414)	6-Month (*n* = 397)	*P* Value^1^
Beliefs About Sex				0.33
Intervention Group	14.06 (2.16)	13.83 (2.20)	13.56 (2.47)	
Control Group	14.09 (2.18)	13.87 (2.37)	13.37 (2.48)	
Beliefs About Abstinence				0.33
Intervention Group	18.00 (4.19)	17.65 (4.68)	18.07 (4.24)	
Control Group	18.29 (3.87)	18.26 (4.35)	18.02 (4.17)	
Perceived Parents Beliefs About Sex				0.64
Intervention Group	14.37 (2.14)	13.95 (2.37)	14.14 (2.27)	
Control Group	14.44 (2.21)	14.09 (2.35)	14.08 (2.24)	
Self-Efficacy for Refusing Sex				0.22
Intervention Group	25.35 (4.12)	24.60 (5.06)	25.28 (4.35)	
Control Group	25.07 (4.30)	24.93 (4.78)	24.69 (4.78)	
Condom Knowledge				0.04
Intervention Group	28.99 (30.33)	32.35 (31.98)	41.37 (35.43)	
Control Group	33.20 (29.69)	31.13 (32.24)	36.55 (31.60)	
Condom Self-Efficacy				0.63
Intervention Group	7.17 (2.56)	7.80 (2.60)	8.28 (2.50)	
Control Group	7.22 (2.53)	7.70 (2.64)	7.89 (2.36)	
Exposure to Risky Situations				0.33
Intervention Group	7.24 (2.78)	7.81 (4.08)	8.01 (4.74)	
Control Group	7.70 (3.88)	7.72 (3.70)	7.92 (4.43)	
HIV/STI Knowledge				0.13
Intervention Group	25.22 (29.68)	26.03 (32.97)	35.69 (35.55)	
Control Group	23.94 (27.40)	24.22 (28.90)	27.41 (32.13)	
Intentions Toward Sex				0.83
Intervention Group	18.52 (4.35)	18.04 (4.52)	19.11 (4.48)	
Control Group	18.49 (4.44)	18.52 (4.42)	18.92 (4.45)	
Expectancies				0.63
Intervention Group	80.61 (15.61)	76.98 (16.34)	77.57 (16.04)	
Control Group	81.02 (16.04)	79.20 (17.62)	79.49 (15.45)	
Communication About Sex Self-Efficacy				0.40
Intervention Group	37.26 (14.48)	37.31 (15.26)	39.38 (15.62)	
Control Group	39.23 (14.30)	37.79 (15.49)	40.69 (15.62)	
Communication About Sex Outcome Expectancy				0.70
Intervention Group	46.66 (9.90)	46.75 (9.80)	48.27 (8.97)	
Control Group	45.53 (9.47)	45.70 (9.15)	47.90 (9.17)	
Communication About Sex				0.19
Intervention Group	14.55 (11.88)	17.14 (15.05)	19.81 (17.93)	
Control Group	14.57 (12.44)	14.78 (12.77)	16.96 (16.61)	
Communication Ability				0.09
Intervention Group	4.39 (1.96)	4.64 (1.86)	4.71 (1.87)	
Control Group	4.66 (1.80)	4.54 (1.86)	4.73 (1.77)	
Parent-Child Connectedness				0.04
Intervention Group	20.00 (5.89)	20.57 (5.13)	20.55 (5.37)	
Control Group	20.79 (4.83)	20.32 (5.11)	20.97 (4.65)	
Parental Monitoring				0.77
Intervention Group	15.40 (4.55)	15.85 (4.39)	16.27 (4.46)	
Control Group	15.54 (4.16)	15.96 (4.15)	16.49 (3.66)	
Intentions and Beliefs About Child Disclosure				0.75
Intervention Group	15.10 (4.33)	15.00 (4.15)	15.16 (4.20)	
Control Group	15.21 (4.05)	14.96 (3.94)	14.95 (3.98)	
Communication Content and Frequency				.001
Intervention Group	2.78 (2.03)	3.25 (2.50)	4.08 (3.05)	
Control Group	2.94 (2.10)	2.84 (2.11)	3.31 (2.59)	

^1^Interaction of Group*Month comparing change in score between groups

### Youth outcomes

Improvements in youth knowledge, communication, and parent-child connectedness were noted (Table 4). Specifically, there was a higher mean score increase in condom knowledge among youth in the intervention group compared to control group (*p* = 0.04). Additionally, intervention group youth had significantly larger improvements in youth reported parent-child connectedness compared to control group youth (*p* = 0.04). Finally, measures of parent-child sexual health communication content and frequency significantly differed with a larger mean difference increase among intervention group youth (*p* = 0.001). No significant differences were found in STI/HIV knowledge, beliefs about sex or abstinence, self-efficacy to refuse sex use a condom. Regarding youth perceptions of parental beliefs, there were no differences in perceived parental beliefs about sex. Rates of sexual activity also remained low throughout the six-month follow-up period with no significant differences in rates of oral, vaginal, or anal sex between youth in the intervention and control groups (Table 4). Less than 2% of youth reported oral, vaginal, or anal sex at baseline. While a small increase was seen during the follow-up period, at six months, less than 3.5% of youth reported being sexually active with no differences seen among the two groups.

**Table 4:** Primary Youth Sexual Behaviors by Condition and Assessment Period

Variable	No *n* (%)	Yes *n* (%)	*P* Value^1^
Baseline (*n* = 508)			
Ever Had Oral Sex			0.50
Intervention Group	250 (98.81%)	3 (1.19%)	
Control Group	247 (98.02%)	5 (1.98%)	
Ever Had Vaginal Sex			0.12
Intervention Group	252 (99.60%)	1 (0.40%)	
Control Group	247 (98.02%)	5 (1.98%)	
Ever Had Anal Sex			1.00
Intervention Group	252 (99.60%)	1 (0.40%)	
Control Group	251 (99.60%)	1 (0.40%)	
1-Month Follow-Up (*n* = 414)			
Ever Had Oral Sex			0.72
Intervention Group	200 (98.04%)	4 (1.96%)	
Control Group	202 (98.54%)	3 (1.46%)	
Ever Had Vaginal Sex			0.28
Intervention Group	202 (99.02%)	2 (0.98%)	
Control Group	199 (97.07%)	6 (2.93%)	
Ever Had Anal Sex			0.22
Intervention Group	200 (98.04%)	4 (1.96%)	
Control Group	204 (99.51%)	1 (0.49%)	
6-Month Follow-Up (*n* = 397)			
Ever Had Oral Sex			0.34
Intervention Group	194 (98.48%)	3 (1.52%)	
Control Group	193 (96.50%)	7 (3.50%)	
Ever Had Vaginal Sex			1.00
Intervention Group	194 (98.48%)	3 (1.52%)	
Control Group	196 (98.00%)	4 (2.00%)	
Ever Had Anal Sex			0.37
Intervention Group	194 (98.48%)	3 (1.52%)	
Control Group	199 (99.50%)	1 (0.50%)	

^1^Chi-square test

### HPV vaccination status

Baseline rates of HPV vaccination were low (initiation = 55.7%, completion = 34.16%) with no significant group differences. No significant differences between the groups were found for vaccination initiation or completion rates by one-month post-intervention. However, by six months post intervention, there was a significant group difference with 70.3% of the intervention group initiating the HPV vaccination series vs. 60.6% for the control group (*p* = 0.02). No difference between the groups was found for HPV series completion at six months. However, by six months, the parents in the intervention group were more likely to intend to give their child all three doses of the HPV series (intervention = 72.13%, control = 54.55%, *p* = .0037). There were no differences in vaccination uptake or completion by gender. Although the percentage of girls in the intervention group that initiated the HPV vaccination by six months is higher than boys (73.02% vs. 66.06%), there was no significant difference by gender in HPV vaccination initiation or completion. At six months, 41.8% of those without vaccination records in the registry reported completing HPV vaccination, compared to 42.3% of those with records listed in the IMMTRAC registry.

## Discussion

The majority of youth are not yet sexually active by 14 years of age which provides a critical point to intervene and bolster parental protective factors and promote HPV vaccination [35]. Evidence suggests that parent-based sexual health interventions designed to delay premature sex work best when delivered to adolescents prior to the onset of sexual activity [40]. Findings from this study provide further evidence of the efficacy of brief parent-based adolescent sexual health interventions for parents of 11–14 year old youth [30, 40]. This study suggests that a student nurse delivered, parent-based sexual health and HPV vaccination intervention is effective at improving parental protective factors including communication frequency, parental involvement, parent-child connectedness, condom knowledge among youth, and HPV vaccination rates among underserved minority communities. Our findings indicate that this brief intervention was able to increase HPV vaccination uptake compared to the control arm. This is critically important given the low vaccination rates found; over 40% of youth had not yet started the HPV vaccine series at baseline.

While the effects on parent-child sexual health communication frequency and HPV vaccination uptake were positive, we found no significant intervention effects on parental monitoring or adolescent sexual activity. Parental monitoring is a well-established parental protective factor for adolescent sexual activity [4, 14]. Despite that the intervention addressed parental monitoring and supervision strategies, baseline rates of monitoring from both the parent and youth measures were already high making it difficult to assess for improvements. Finally, very few youth initiated sexual activity during the study period making it difficult to detect group differences at six-months.

Given the intervention effects on communication and connectedness and the positive mediating effect of parent-child sexual health communication [30] and connectedness [23], it was surprising to see little evidence of intervention impact on adolescent sexual behaviors. However, this is likely due to the low levels of sexual activity at baseline and follow-up among this sample of adolescents making it difficult to detect behavioral changes on sexual behavior. A recent meta-analysis found that intervention effects on adolescent sexual behavior are stronger when the intervention is longer, developmentally and culturally tailored to the specific target groups, and includes components for both parents and youth [40]. Therefore, a longer intervention follow-up period and inclusion of youth with greater variability in the onset and frequency of sexual behavior may increase the ability to assess intervention effects on delay of sex and condom use [12].

While both parents and youth reported improvements in communication frequency, there was discordance among the parent-child dyads regarding connectedness, an important factor in parent-youth measurement [41]. While there were no significant intervention effects among parents, there was a significant improvement in youth reported connectedness among the intervention group. Additionally, while no difference in connectedness was found among parents, there was a significant improvement in youth reports of parental involvement between the intervention and control groups.

Notably, this intervention can be delivered in approximately 45 min with two brief follow-up calls. Therefore, the ease of delivery may improve access to and uptake of parent-based interventions. Finding creative ways of addressing the burden of participation for parents such as delivering interventions where parents are at in their daily routine may increase the reach into underserved communities [12]. Other venues that should be explored are school pick-up lines common in some communities, school registration nights, and school open-house events. Delivery of interventions in the clinic setting and online also demonstrate efficacy [13, 36]. A longer workplace intervention has also shown promise and may suggest the need for assessing the efficacy of delivering a brief parent-based adolescent sexual health intervention in the workplace [32].

The efficacy of this intervention also suggests that student nurses are effective interventionists for parent-based adolescent sexual health among high-risk, underserved populations. In addition to serving the public health goals of promoting adolescent sexual health and improving HPV vaccination rates, this delivery format allows for action oriented, community-based health promotion learning for student nurses. This is particularly important given the need to address person-centered care and population health as core competencies in undergraduate nursing education.

Despite the promising findings of the intervention, there are study limitations that should be considered when interpreting the findings. Specifically, this study only followed parents and youth for six-months after the intervention. Therefore, longer term sustainability of intervention effects are not known. As well, very few youth in the sample were sexually active at baseline or at the six-month follow-up making detection of intervention effects on sexual behaviors not possible. A longer follow-up period may assist in assessing intervention effects on adolescent sexual activity. The sample was primarily comprised of youth of color and therefore may not be generalizable to populations other than Black and Latino youth and parents. As well, while some fathers and grandparents participated, the large majority of caregivers were mothers with samples too small to examine findings specifically among fathers or grandparents. Further, this intervention may need to be modified for implementation in other countries and cultures. Lastly, the primary outcomes were self-report. Therefore, social desirability in adolescent reports of sexual behavior cannot be ruled out. Future research should include the use of biomarkers (i.e., STI point-of-care test) as an additional measure to adolescent self-report of sexual behavior.

## Conclusion

Adolescence is a critical time to influence the adoption of lifelong protective behaviors and overall well-being [27]. Therefore, we must continue to research novel methods and strategies to addressing persistent sexual and reproductive health disparities among adolescents, particularly youth of color. Nurses are on the frontlines of healthcare, both highly competent in caring for complex health conditions among individuals and being uniquely positioned in community settings to bridge the gap between health promotion science and implementation across hard-to-reach communities [29]. Student nurses are effective parent-based adolescent sexual health interventionists. Therefore, investing in the training and utilization of student nurses as facilitators of prevention interventions serves our communities and lays the groundwork for effective nursing practice among the largest segment of the healthcare workforce. Further research is needed to examine the impact of delivering evidence-based prevention interventions in the community on the learning satisfaction and clinical competencies of student nurses.

## Supplementary Information

**Additional file 1.**

## Data Availability

The datasets during and/or analyzed during the current study available from the corresponding author on reasonable request.

## Abbreviations

HPV: Human papillomavirus
FTT: Families Talking Together
