# Supplementary material for: Effects of a randomized controlled trial of a brief, student-nurse led, parent-based sexual health intervention on parental protective factors and HPV vaccination uptake
Source: BMC Public Health. 2021 Mar 24;21:585. doi: 10.1186/s12889-021-10534-0 (PMC7992324; doi:10.1186/s12889-021-10534-0)
Supplement: Supplementary file 1 — Additional file 1. [file 12889_2021_10534_MOESM1_ESM.docx]

**Sensitivity Analysis for FTT+HPV Study**

Repeated measures analyses with linear mixed models were used to conduct a sensitivity analysis to assess the impact of missing data through loss to follow-up. These models contained three effects: **Month**, which tests for a change in the overall mean outcome score between visit months (0, 1, and 6); **Miss**, which compares the overall mean outcome score for those that were lost to follow-up and those that completed the study, and the interaction of Month and Miss (**Miss*Month**). This latter effect is of primary interest in the sensitivity analysis (p value in bold print) in that it tests if the change in the outcome score varies between those that were lost to follow-up and those that completed the study. The linear mixed models include all participants with one or more observations in the analysis, with estimates of change in the outcomes based on all observable data. These models did not show evidence of a difference for change in outcome scores due to loss to follow-up (all p > .18).

**Parent Outcome**

| **Model Information** | |
| --- | --- |
| **Data Set** | WORK.THREEA |
| **Dependent Variable** | **Frequency of Communication** |
| **Covariance Structure** | Compound Symmetry |
| **Subject Effect** | Q238 |
| **Estimation Method** | REML |
| **Residual Variance Method** | Profile |
| **Fixed Effects SE Method** | Kenward-Roger |
| **Degrees of Freedom Method** | Kenward-Roger |

| **Type 3 Tests of Fixed Effects** | | | | |
| --- | --- | --- | --- | --- |
| **Effect** | **Num DF** | **Den DF** | **F Value** | **Pr > F** |
| **Month** | 2 | 890 | 24.84 | <.0001 |
| **Miss** | 1 | 769 | 0.06 | 0.8011 |
| **Miss*Month** | 1 | 970 | 0.58 | **0.4450** |

**Parent Outcome**

| **Model Information** | |
| --- | --- |
| **Data Set** | WORK.SIXA |
| **Dependent Variable** | **Parental Involvement** |
| **Covariance Structure** | Compound Symmetry |
| **Subject Effect** | Q238 |
| **Estimation Method** | REML |
| **Residual Variance Method** | Profile |
| **Fixed Effects SE Method** | Kenward-Roger |
| **Degrees of Freedom Method** | Kenward-Roger |

| **Type 3 Tests of Fixed Effects** | | | | |
| --- | --- | --- | --- | --- |
| **Effect** | **Num DF** | **Den DF** | **F Value** | **Pr > F** |
| **Month** | 2 | 844 | 6.24 | 0.0020 |
| **Miss** | 1 | 683 | 0.37 | 0.5440 |
| **Miss*Month** | 1 | 912 | 0.68 | **0.4095** |

**Youth Outcome**

| **Model Information** | |
| --- | --- |
| **Data Set** | WORK.THREEY |
| **Dependent Variable** | **Condom Knowledge** |
| **Covariance Structure** | Compound Symmetry |
| **Subject Effect** | Q278 |
| **Estimation Method** | REML |
| **Residual Variance Method** | Profile |
| **Fixed Effects SE Method** | Kenward-Roger |
| **Degrees of Freedom Method** | Kenward-Roger |

| **Type 3 Tests of Fixed Effects** | | | | |
| --- | --- | --- | --- | --- |
| **Effect** | **Num DF** | **Den DF** | **F Value** | **Pr > F** |
| **Month** | 2 | 850 | 13.85 | <.0001 |
| **Miss** | 1 | 672 | 0.85 | 0.3571 |
| **Miss*Month** | 1 | 906 | 1.27 | **0.2595** |

**Youth Outcome**

| **Model Information** | |
| --- | --- |
| **Data Set** | WORK.SIXY |
| **Dependent Variable** | **Parent-Child Connectedness** |
| **Covariance Structure** | Compound Symmetry |
| **Subject Effect** | Q278 |
| **Estimation Method** | REML |
| **Residual Variance Method** | Profile |
| **Fixed Effects SE Method** | Kenward-Roger |
| **Degrees of Freedom Method** | Kenward-Roger |

| **Type 3 Tests of Fixed Effects** | | | | |
| --- | --- | --- | --- | --- |
| **Effect** | **Num DF** | **Den DF** | **F Value** | **Pr > F** |
| **Month** | 2 | 837 | 0.64 | 0.5249 |
| **Miss** | 1 | 670 | 1.87 | 0.1715 |
| **Miss*Month** | 1 | 897 | 1.74 | **0.1873** |

**Youth Outcome**

| **Model Information** | |
| --- | --- |
| **Data Set** | WORK.NINEY |
| **Dependent Variable** | **Communication Content and Frequency** |
| **Covariance Structure** | Compound Symmetry |
| **Subject Effect** | Q278 |
| **Estimation Method** | REML |
| **Residual Variance Method** | Profile |
| **Fixed Effects SE Method** | Kenward-Roger |
| **Degrees of Freedom Method** | Kenward-Roger |

| **Type 3 Tests of Fixed Effects** | | | | |
| --- | --- | --- | --- | --- |
| **Effect** | **Num DF** | **Den DF** | **F Value** | **Pr > F** |
| **Month** | 2 | 865 | 24.63 | <.0001 |
| **Miss** | 1 | 707 | 0.25 | 0.6139 |
| **Miss*Month** | 1 | 927 | 1.60 | **0.2060** |
